# Uterus Transplantation as a Therapy Method in Mayer-Rokitansky-Küster-Hauser Syndrome

**DOI:** 10.7759/cureus.6333

**Published:** 2019-12-09

**Authors:** Nikolaos Georgopapadakos, Arezina Manoli, Georgia Passia, Panagiotis N Skandalakis, Dimitrios Filippou

**Affiliations:** 1 Anatomy and Surgical Anatomy, Medical School of National and Kapodistrian University of Athens, Athens, GRC; 2 Otolaryngology, Medical School of National and Kapodistrian University of Athens, Athens, GRC; 3 Surgical Anatomy, National and Kapodistrian University of Athens, Athens, GRC; 4 Surgery, Medical School of National and Kapodistrian University of Athens, Athens, GRC

**Keywords:** mrkh, vaginal, aplasia, agenesia, vaginal transplant, anatomy, anatomical variants, transplantation

## Abstract

The Mayer-Rokitansky-Küster-Hauser (MRKH) syndrome is the most common cause of uterine aplasia (underdevelopment or absence) at a frequency estimated to be worldwide of 1/4500 births of new-born female infants.

This is a literature review aiming to determine the sufficiency of the uterine transplantation (UTx) method as a therapeutic protocol for the MRKH syndrome.

Online searches were carried out in PubMed, Embase, CINAHL and Google scholar databases, during January and February 2019. The search included a combination of the various terms (see key words) as well as a combination of these terms in Greek and English so as to identify and display articles that would be as close as possible to the subject of research.

The online search yielded 95 articles. Eighty-five of these were considered as eligible and possible sources from the title and abstract presented but later were excluded, whereas 10 of them were selected to be included in the literature review. The literature review results showed that two therapeutic methods that are now successfully applied are the Vecchietti method and the Davydov method, which is the latest and less invasive technique but with equally if not improved immediate results. However, this treatment is not adequate to satisfy or provide a solution for the reproduction requirements of this patient group. The UTx proved sufficient.

Although uterus transplant could be considered the ideal solution for the management of infertility and the satisfaction of the reproductive and sexual needs of women with MRKH syndrome, since the first successful pregnancy after uterine transplantation is a reality in the recent years, it is early days to be considered as a safe mode of management.

## Introduction and background

The Mayer-Rokitansky-Küster-Hauser (MRKH) syndrome is a rare genetic condition where the vagina and the uterus are underdeveloped or (as in most cases) not developed at all [1]. It is the most common cause of uterine aplasia at a frequency estimated to be worldwide of 1/4500 births of new-born female infants [2]. The exact cause of MRKH syndrome is still unknown as the person’s karyotype is normal (46, XX) and any chromosomal abnormalities are extremely rare. In most cases, diagnosis is achieved at 15-21 years of age due to persistent amenorrhoea [1].

Diagnosis of MRKH requires clinical examination and ultrasound imaging techniques in order to confirm the existing uterine and vaginal aplasia and the existence of normal ovaries. In approximately 40% of the diagnosed cases abnormalities of the urinary system (solitary kidney duplicated ureters etc.) are detected, while 15% of diagnosed cases present skeletal abnormalities [3]. MRKH is connected with two serious problems for women who suffer from it. The first is gestation failure due to lack of uterus and the inability to complete sexual contacts due to lack of vagina, which causes tremendous psychological problems to the patient [4].

The uterus, the vagina and the ovaries are essential organs of the female reproduction system. However, there are reported cases of females born with congenital abnormalities in their genitals, such as aplasia of the vagina and the uterus, presented in the MRKH syndrome. In the case of MRKH syndrome, a woman with normal karyotype, is born without a uterus or has only two hypoplastic horns of the uterus and the vaginal canal is noticeably shortened (3/4 of the vagina is absent), mainly due to the failure of the Müllerian duct to develop [5]. The ovaries are perfectly normal, since they are developed from other embryological structures, and for this reason these girls will exhibit normal development, namely their sex-related secondary characteristics (normal external genital organs, normal breasts and normal hair-growth in the pubis and armpits), but they fail to present menstruation [5]. Additionally, up to 40% of these cases present coexisting congenital abnormalities of the urinary system such as the absence of a kidney or the presence of only one kidney in the lower abdomen (pelvic kidney) or other abnormalities [6].

MRKH syndrome is genetically linked to the inheritance of an autosomal dominant gene with inadequate penetrance and inconstant expressivity, resulting to a high difficulty in the identification of the underlying causing mechanisms. Classification of the MRKH syndrome is based on the variability of the inheritance, penetrance and expressivity patterns [7]. There are two MRKH subtypes: type 1, in which genital structures deriving from the Müllerian duct, such as the cervix, the upper vagina and uterus, are underdeveloped, and type 2, where the same reproductive structures are disturbed, but additional malformations of other body parts are also presented, with renal and skeletal malformations and Müllerian Renal Cervical Somite (MURCS) to be the most common [7]. The primary reasons of MRKH syndrome are still under research, but numerous contributing genes have been examined as the potential cause for the development of the syndrome. However, the results of most of these studies dismiss genes of being the contributing factors in MRKH, but so far, the WNT4, HNF1B, and LHX1 genes have been linked with MRKH inheritance [8]. The purpose of this paper is to make an as much as possible complete record and the usefulness of uterus transplant as a possible method of treatment and restoration of the MRKH syndrome.

## Review

Material and methods

Online searches were carried out in PubMed, Embase, Cinahl and Google scholar databases, during January and February 2019. The search included a combination of the various terms (e.g., MRKH, uterus transplantation, Vecchietti neovagina, infertility treatment, anatomical variants, etc.) as well as a combination of these terms in English so as to identify and display articles that would be as close as possible to the subject of research. The selection process for the articles included in the literature review is shown in Table 1.

**Table 1:** Selection process for the articles included in the literature review

Online search in the Pubmed, EMBASE, Cinahl & Google Scholar	
Search results (n = 95)	
Review of articles based on title & abstract	
Accepted for further review and analysis (n = 10)	Exclusion due to noncompliance with inclusion criteria (n = 85) – Non-accessible (full text not available): 45; Disputed results: 25; Repeated articles: 15
Review based on inclusion criteria	
Articles included in review (n = 10)	Exclusion due to noncompliance with inclusion criteria (n = 0)

The criteria for article inclusion used to complete the systematic review of the literature are:

• The articles included in the research should be written in English or translated into this language ​​from their original version.

• The included articles should have been published by official scientific organisations.

• Their release dates should be between 2009 and 2019.

• Any type of studies (clinical, case, etc.) could be included but their results should have been confirmed by similar studies.

Results

The online search yielded 95 articles. Eighty-five of these were considered as eligible and possible sources from the title and abstract presented but later were excluded, whereas 10 (Table 2) of them were selected to be included in the literature review. The most common reason to exclude an article from any further consideration was the lack of a combination of the original data and the inability to access the full article.

**Table 2:** Literature review results of uterus transplantation as method of treatment and restoration of the MRKH syndrome MRKH: Mayer-Rokitansky-Kuster-Hauser

Author and date	Title	Type of research	No of participants	Conclusion
Diaz-Garcia et al., 2012 [9]	Uterine transplantation research: laboratory protocols for clinical application.	Review study		Recent advances in the field of solid organ transplantation and experimental Uterine transplantation provide a favorable and safe background in a scenario in which a human clinical uterine transplantation trial can take place.
Ozkan et al., 2013 [10]	Preliminary results of the first human uterus transplantation from a multiorgan donor.	Case study	1	The longest-lived transplanted human uterus case with acquirement of menstrual cycles is studied and described.
Brännström et al., 2014 [11]	First clinical uterus transplantation trial: A six month report.	Prospective observational study	9	This study shows that a live-donor uterine transplantation procedure has a low risk despite extended surgery duration. The report of the first successful human uterine transplantation case, defined as a live birth from a transplanted human uterus, has yet to be published.
Brännström et al., 2015 [12]	Live birth after uterus transplantation.		1	This report is a proof-of-concept for uterus transplantation as a treatment for uterine factor infertility. Furthermore, the results show the feasibility of live uterus donation, even from a postmenopausal donor.
Johannesson et al., 2015 [13]	Uterus transplantation trial: 1-year outcome.	Prospective observational study	9	The results of the present study demonstrate long-term uterine viability and function after live-donor uterine transplantation. Asymptomatic rejection episodes can be detected by cervical tissue biopsies and resolved by temporary addition of glucocorticoid treatment.
Brännström et al., 2017 [14]	Uterus transplantation and beyond. Journal of materials science.	Review study	11	Classical uterine transplantation procedure, with transplantation from live or deceased donors, will only stay as the predominant infertility treatment for Absolute uterine factor infertility women for one or two decades, since creation of bioengineered uterus may enter the clinical arena in the future.
Castellón et al., 2017 [15]	The history behind successful uterine transplantation in humans.	Review study	25	Uterus transplantation has demonstrated its potential as a highly effective treatment for infertility due to congenital or acquired uterine absence, especially in patients with Mayer Rokitansky Kuster Hauser Syndrome.
Suganuma et al., 2017 [16]	Uterus transplantation: Toward clinical application in Japan.	Review study	24	In total, 42 women worldwide have received transplanted wombs and 11 babies have been born up as a result until May 2017. It cannot be denied that uterine transplantation is still under development as a reproductive medicine and organ transplant procedure.
Zaami et al., 2017 [17]	Ethical and medico-legal remarks on uterus transplantation: may it solve uterine factor infertility?	Review study		Uterus transplant cannot be regarded as a life-saving procedure, but rather a method to restore woman ability to procreate, when lost, thus improving her quality of life. Uterus transplant is a complex surgical procedure and presents significant health threats.
Chmel et al., 2018 [18]	The Interest of Women with Mayer-Rokitansky-Kuster-Hauser Syndrome and Laparoscopic Vecchietti Neovagina in Uterus transplantation.	Prospective observational study	50	Nearly two-thirds of the Mayer Rokitansky Kuster Hauser syndrome study group of women with surgically created neovaginas were interested in uterus transplantation and motivated to undergo this method of absolute uterine factor infertility treatment.

Discussion

In the past, many surgical and laparoscopic methods have been used to restore the vagina by creating a neovagina. An operation such as the neovagina opening is only performed after a diagnosis is performed by both radiographic and laparoscopic techniques, and involves a very brief and abridged opening and formation of a 9-11 cm long vagina from the skin of the vulva itself [19]. Two methods are now successfully applied, the Vecchietti method and the Davydov method, which is the latest and less invasive technique but equally if not improved immediate results [20, 21]. However, this treatment is not adequate to satisfy or provide a solution for the reproduction requirements of this patient group. People with other genital anomalies due to the Müllerian ducts underdevelopment, have no real reproductive benefits from these surgeries and exhibit high failed implantation and miscarriage rates [22].

A 2000 report of the successful uterine transplantation (UTx) to a 32-year-old woman, diagnosed with MRKH, made it a desirable treatment method [23]. Previous studies carried out in laboratory animals had proved the sufficiency of the UTx method (animal studies in mice, rats, rabbits, larger animals such as pigs and sheep, and non‐human primates such as monkeys and baboons) [24-29]. UTx has validated its capability as a greatly effectual solution for the infertility problems presented due to uterine agenesia or aplasia in humans as well [9, 10, 15].

Since 2011 and even earlier, reports of women born with uterus aplasia and diagnosed with MRKH syndrome that have undergone UTx managed not only to retain the transplanted organ but even went on to produce offspring carrying out normal pregnancies and giving birth to healthy children. In total, 42 women worldwide have received transplanted wombs and 11 babies have been born up as a result until May 2017 [16]. The age of both the donor and the recipient, as well as whether the donor is alive or deceased, seem to be important factors for the successful outcome of the transplant. Many of published trials point out that the ideal recipients of the uterus transplant should not be any older than 35-40 years of age whereas the donors could be older and even post-menopausal, with the higher age limit to be 55-65 years old [11, 12, 14].

While womb transplants appear to be ideal for women suffering from MRKH, the procedure is still considered experimental, since no therapeutic protocols have been as yet established and the ethical and medical concerns are still under investigation [16, 17, 30]. Screening of both suitable donors and recipients is still based on the conclusions of previous studies and on institutional protocols, which mainly include the physical state of the donor, the absence of infection or tissue damaging diseases such as cancer [14]. Additionally in women with MRKH, the type of uterus complication is also a contributing factor as women with underdeveloped or absent uterus should undergo vaginal reconstruction prior to transplantation surgery [9, 14]. Moreover, further serious complications presented post operational on the recipients include infections and urinary tract-related complications, thrombosis and haematoma development [9, 10].

According to research findings, asymptomatic rejection episodes can occur but are easily detected by cervical tissue biopsies and are temporarily treated with additional glucocorticoid treatment. Additionally, undesirable side effects of immunosuppressant, used to minimise organ rejection possibility, could be the main reason of the development of preeclampsia and the high-risk pregnancy in women that proceed with reproduction and pregnancy after the transplantation [30]. Methods for the control of treatment-resistant rejection of the transplanted uterus during pregnancy are still necessary for women that have undergone UTx even when prior to the pregnancy no signs of organ rejection are present, however, such measurements are still under development and not as yet perfected [12].

## Conclusions

Although UTx could be considered the ideal solution for the management of infertility and the satisfaction of the reproductive and sexual needs of women with MRKH syndrome, it is early days to be considered as a safe mode of management. The success for which the scientific team faced technical obstacles that seemed unsurpassed is a real milestone for the in vitro fertilisation and assistive reproductive technology field, but due to the variable possible complications of the procedure itself and for the post operational period, we are still far from incorporating this method into therapeutic protocols targeted for infertility treatment for women with congenital uterus aplasia or agenesia. The future will show if this intervention is to be applied on a large scale.
